# Eine seltene Ursache für akute Herzinsuffizienz? Aorta-rechtsatriale Fistel!

**DOI:** 10.1007/s00108-023-01538-6

**Published:** 2023-06-05

**Authors:** Benedikt Marahrens, Dominique Sebastian Petrus

**Affiliations:** Zentrum für Innere Medizin 1 – Kardiologie, Universitätsklinikum Brandenburg, Medizinische Hochschule Brandenburg, Hochstr. 29, 14770 Brandenburg, Deutschland

**Keywords:** Perikardpatch, Aortenklappe, Dyspnoe, Echokardiographie, Ödeme, Pericardial patch, Aortic valve, Echocardiography, Dyspnea, Edema

## Abstract

**Hintergrund:**

Akute Herzinsuffizienz mit Symptomen wie Dyspnoe und Ödemen kann diverse Ursachen haben, in seltenen Fällen auch kardiale Fisteln. Wir präsentieren einen Fall von subakuter Herzinsuffizienz verursacht durch eine erworbene Fistel zwischen Aorta und rechtem Vorhof.

**Fallbericht:**

Ein 48-jähriger Patient stellte sich mit zunehmender Belastungsdyspnoe und Ödemen der unteren Extremitäten seit circa 4 Wochen in der Klinik vor. Echokardiographisch konnte eine Fistel zwischen Aorta und rechtem Atrium nachgewiesen werden. Zum Verschluss wurde er an ein thoraxchirurgisches Zentrum überwiesen.

**Video online:**

Die Online-Version dieses Beitrags (10.1007/s00108-023-01538-6) enthält fünf Videos.

## Abstract (en)

**Background:**

Acute heart failure with symptoms such as dyspnea and edema has various causes. In rare cases cardiac fistulas can cause acute heart failure. Herein we present a case of subacute heart failure due to an acquired fistula between the aorta and right atrium.

**Case report:**

A 48-year-old male was referred to the emergency room with increasing dyspnea on exercise and pitting edema of the lower extremities starting approximately 4 weeks previously. Echocardiographic workup showed an aorta-to-right atrium fistula. The patient was referred to a cardiothoracic surgery center for closure of the fistula.

## Anamnese

Ein 48-jähriger Mann wurde von seinem Hausarzt in die Notaufnahme eingewiesen, weil er seit etwa 4 Wochen unter zunehmender Belastungsdyspnoe litt. Diese sei in den letzten Tagen bereits bei geringster Belastung aufgetreten. Er berichtet, dass einige Angehörige seiner Familie Herzprobleme hätten, konnte diese Angabe allerdings nicht weiter spezifizieren. Husten, Fieber, Brustschmerzen und andere Symptome wurden vom Patienten verneint.

## Befunde

In der Rettungsstelle war der Patient normoton mit Blutdruck 125/65 mmHg, Puls 112/min, 95 % unter Raumluft und Temperatur 36,5 °C.

Die Laboruntersuchungen ergaben einen NT-proBNP-Wert von 2215 pg/ml. Die übrigen Laborergebnisse lagen im Normalbereich oder nur geringfügig außerhalb der Norm. Die COVID-19-PCR war negativ. Das EKG zeigte einen Indifferenztyp mit tachykardem Sinusrhythmus ohne Erregungsrückbildungsstörungen.

**Figure 1:**
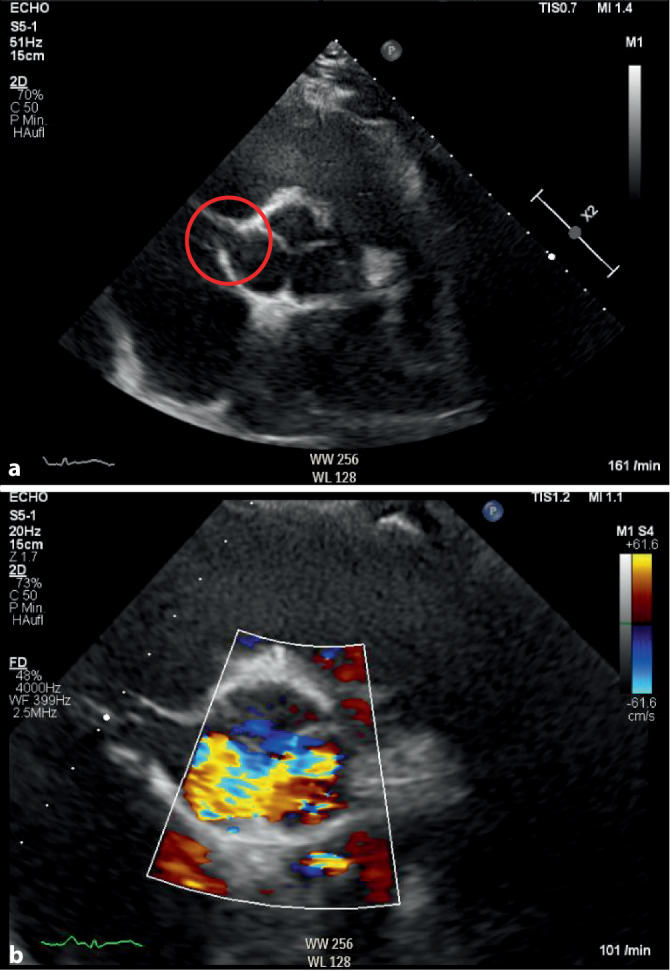

Es erfolgte eine computertomographische (CT) Angiographie des Thorax zum Ausschluss einer Lungenembolie und weiterer pulmonaler Ursachen. In der transthorakalen Echokardiographie (TTE) sahen wir eine trikuspide Aortenklappe mit turbulenten Blutströmen in der Aortenwurzel und eine Struktur, die sich wie eine Fistel zwischen Aorta und rechtem Atrium präsentierte (Abb. 1 sowie Video 1 und Video 2).

## Diagnose

**Figure 2:**
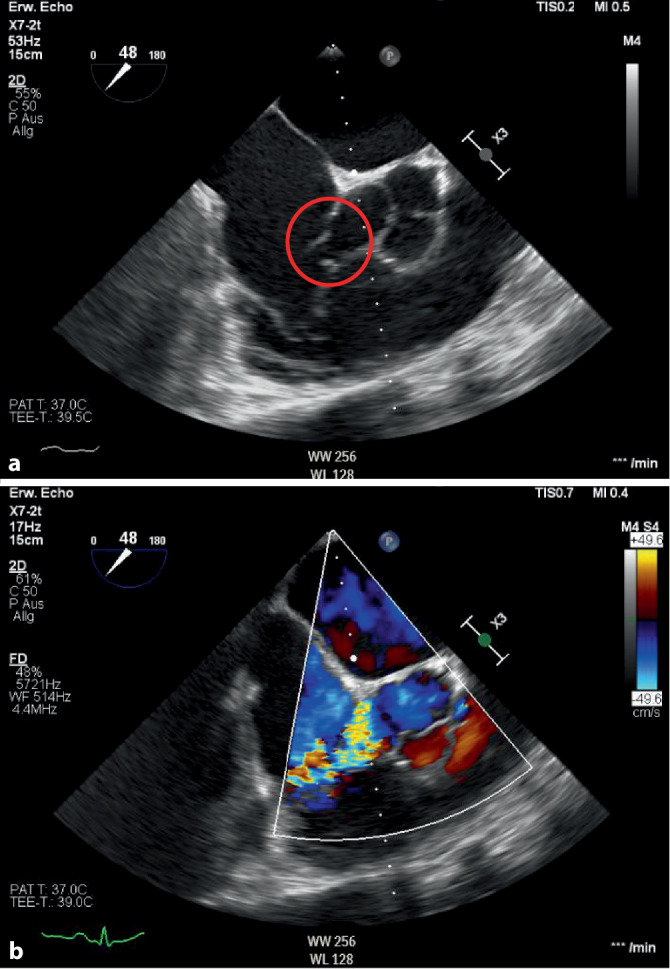

**Figure 3:**
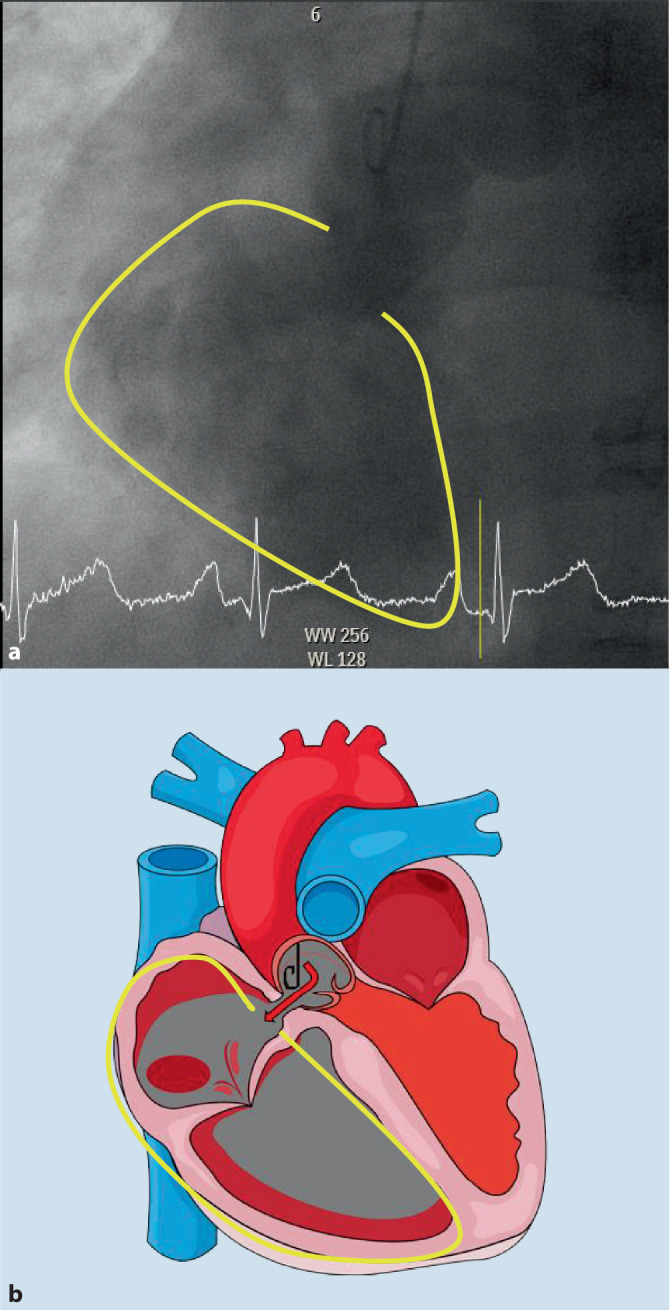

– In der transösophagealen Echokardiographie (TEE) konnten wir die Fistel zwischen Aorta und rechtem Vorhof mit einem Strahl knapp oberhalb der Trikuspidalklappe bestätigen (Abb. 2 sowie Video 3 und Video 4).

– Um den Koronarstatus des Patienten vor einer thoraxchirurgischen Operation zu beurteilen und die Lokalisation der Fistel weiter zu untersuchen, führten wir eine perkutane transluminale Angiographie (PTA) durch. Dabei zeigte sich ein Kontrastmittelfluss von der Aorta direkt in den rechten Vorhof (Abb. 3 sowie Video 5). Wir konnten den Führungsdraht direkt von der Aorta in den rechten Vorhof einführen, wobei der Druck, gemessen über den Katheter, abrupt abfiel.

## Therapie und Verlauf

Der Patient wurde dem nahegelegenen Zentrum für Thoraxchirurgie vorgestellt und kurz darauf zur Patchplastik der Fistel dorthin verlegt. Aufgrund der Nähe der Fistel zur Aortenklappe konnte die Patchplastik nur mit weniger straffen Nähten umschlagen werden. Andernfalls hätte der Patient sich einem zusätzlichen Aortenklappenersatz unterziehen müssen. Intraoperativ zeigte sich als Ursache der Fistel ein rupturiertes Aneurysma des Sinus Valsalva. Postoperativ zeigte sich der Rechtsshunt zwischen Aorta und rechtem Vorhof stark reduziert, aber nicht vollständig verschwunden. Im Verlauf entwickelte der Patient einen AV-Block III°, sodass ihm ein Herzschrittmacher implantiert werden musste. Etwa eine Woche nach der Operation wurde der Patient entlassen und es wurde eine Rehabilitation geplant.

## Diskussion

Akute Herzinsuffizienz kann durch verschiedene Entitäten, wie ein akutes Koronarsyndrom oder eine Endokarditis, verursacht werden [1]. Eine sehr viel seltenere Ursache für eine akute Herzinsuffizienz ist eine erworbene Fistel, beispielsweise zwischen Aorta und rechtem Atrium (ARAF) – wie in diesem Fallbericht beschrieben [2]. Die meisten Fisteln bestehen ab der Geburt oder frühen Kindheit und verursachen oft dramatische Veränderungen der kardialen Hämodynamik [3]. Erworbene Fisteln können auch zu hämodynamischen Veränderungen, wie Volumenüberlastung, führen [2].

Eine erworbene ARAF wurde erstmals 1961 beschrieben und tritt am häufigsten nach Trauma, einschließlich stumpfer Verletzungen, penetrierender Traumata und Operationen, wie Aortenklappenersatz, auf [4, 5]. Andere Ursachen sind unter anderem infektiöse Endokarditis und Aortendissektion [6, 7]. Eine weitere sehr viel seltenere Ursache für diese Art von Fistel ist eine Ruptur eines Aneurysmas oder Pseudoaneurysmas des Sinus Valsalva [8].

Es gibt verschiedene Möglichkeiten, eine ARAF zu versorgen. Wie in diesem Fall beschrieben, kann sie durch einen thoraxchirurgischen Eingriff mit einem Perikardpatch oder künstlichem Material repariert werden, wie es auch bei anderen Fistelreparaturen häufig durchgeführt wird [9]. Bei diesem Therapieansatz kann die Fistel am besten dargestellt und untersucht werden, er stellt allerdings auch die invasivste Methode dar. Die Versorgung der Fistel kann sich auch hier als schwierig erweisen, je nach genauer Lage der Fistel, wie auch im hier beschriebenen Fall. Ein weiterer, weniger invasiver, Therapieansatz ist der katheterassistierte Verschluss unter PTA durch einen Schirm aus Kunstmaterial, über den in einigen Fallberichten berichtet wird [10]. Dieser Ansatz erfordert jedoch einen erfahrenen Untersucher und die Fistel muss sich an einer Stelle befinden, die für einen Katheter gut zugänglich ist.

## Fazit für die Praxis

- Patienten mit Symptomen einer akuten Herzinsuffizienz sollten schnellstmöglich eine TTE erhalten, denn so können auch seltene Ursachen wie die ARAF zügig erkannt werden. Als weiterführende Diagnostik können TEE, CT und PTA hilfreich sein.
- Aufgrund der diversen, unterschiedlich invasiven Versorgungsmöglichkeiten einer ARAF empfehlen wir, diese Fälle individuell in einem interdisziplinären Team (Herzteam) zu besprechen, wie es auch bei der Entscheidung über die Vorgehensweise bei Herzklappenreparaturen praktiziert wird.

## Supplementary Information








